# Surviving COVID-19: Biopsychosocial Impacts, Death Anxiety, and Coping Strategies

**DOI:** 10.3390/vaccines11030705

**Published:** 2023-03-20

**Authors:** Amina Muazzam, Faiqa Naseem, Muneeba Shakil, Anna Visvizi, Jolanta Klemens

**Affiliations:** 1 Department of Psychology, Lahore College for Women University, Jail Road, Lahore 54000, Pakistan; 2 Department of Humanities, COMSATS University Islamabad, Lahore Campus, 1.5 KM Defence Road, Off Raiwind Road, Lahore 54000, Pakistan; 3 Institute of International Studies (ISM), SGH Warsaw School of Economics, Al. Niepodległości 162, 02-554 Warsaw, Poland; 4 Effat College of Business, Effat University, Jeddah 21551, Saudi Arabia; 5 PSYCHOMedical, ul. W. Broniewskiego 39, 43-300 Bielsko-Biała, Poland

**Keywords:** COVID-19, COVID-19 survivors, death anxiety, coping strategies, the BPS model

## Abstract

As the COVID-19 pandemic erupted, attempts to contain the spread of the virus took two concurrent forms, including mobility restrictions (aka lockdowns) and the race to produce a vaccine. However, it is quite striking that, amidst both the lockdown and the race to produce a vaccine, the question of how COVID-19 survivors/patients coped with the disease has not received the degree of attention it deserved. To navigate this issue, we employed a sample consisting of 100 COVID-19 survivors; this paper explores the relationship between the biopsychosocial (BPS) impacts of COVID-19, death anxiety, and coping strategies. In this context, the mediating role of death anxiety is placed in the spotlight. The analysis reveals a significant positive association between the BPS impact of COVID-19 and death anxiety and a significant negative association between death anxiety and coping strategies among COVID-19 survivors. Thus, death anxiety mediates the relationship between the BPS impact and the coping strategies that COVID-19 survivors adopt. Given the general recognition of the validity of the BPS model in contemporary medical science and practice, a thorough examination of COVID-19 survivors and their experiences related to surviving is necessary to match the challenges of today, including the increased probability of pandemics.

## 1. Introduction

From 2019 to 2020, and later, attempts to curb the global spread of the COVID-19 pandemic included top-down approaches, including restrictions to mobility (aka lockdown) and a race to produce a vaccine. While the lockdown and its implications, (e.g., psychological, health (Mukaetova-Ladinska and Kronenberg, 2021), social, political, and economic implications [1,2,3,4,5] have been broadly discussed in the literature, the complex intricacies of the vaccine race still need to be comprehended [6]. To some extent, these debates seem to have obscured the question of how COVID-19 survivors have coped with the disease and its implications.

Admittedly, the surge in COVID-19 cases throughout 2020 has led to elevated numbers of hospitalizations globally. They have also caused stress, anxiety, and fear across societies worldwide. The spread of COVID-19 has been linked to a dramatic increase and/or deterioration in mental health disorders across all age groups. An increase in panic attacks, anxiety, and depression was recorded. Clearly, however, the impact of COVID-19 has not been limited to mental health. The physical and social well-being of societies were also at stake. The long-term implications of COVID-19-induced lockdowns related to remote learning and remote work are still not yet fully understood [6,7]). The same applies to COVID-19 survivors and their families. This broader way of viewing COVID-19 and its implications, i.e., a view that goes beyond the focus on the symptoms and, thus, the body, and recognizes the need to examine COVID-19 through the lens of the broader realm of the survivor’s family and society, is congruent with the biopsychosocial (BPS) model of medicine [8].

It is widely accepted that COVID-19 has had an unprecedented impact on societies and individuals around the world and that the implications spanned the body, mind, and social structures. There is a growing consensus that COVID-19 and the multi-scalar implications for survivors and their families require a broad, comprehensive explanatory framework, such as the BPS model [9,10,11,12]. There is also a growing body of literature confirming the impact of the modern means of communication, at the local, regional, national, and global levels on (frequently aggravated) perceptions of fear, anxiety, and stress among society members [13,14,15]. Regardless of the progress made in research, relatively little has been written about how those who tested positive for COVID-19 experienced and coped with the disease, not only in terms of bodily symptoms but, most importantly, the psychological and social implications thereof [16,17,18].

Recognizing the salience of the problem, to bring it to the attention of a broader audience, this paper focuses explicitly on the experiences of COVID-19 survivors and their coping strategies to demonstrate the broader implications of COVID-19. The remainder of this paper is structured as follows. The relevance of the BPS model for the examination of the COVID-19 experience is outlined in Section 2. Section 3 elaborates on the research method and materials. The findings, discussion, and limitations of this study are presented in Section 4. Conclusions and future research directions are presented in Section 5.

## 2. Literature Review

### 2.1. Contextual Models to Examine COVID-19 and Its Implications

Throughout the decades, if not millennia (think of Hippocrates and Asclepius), there have been many discussions on how to conceptualize illness, disease, and wellbeing, as well as corresponding discussions about defining what constitutes the norm (and aberrations from the norm), ways to treat an illness/disease, and so on. To a large extent, the modern debate on how to conceptualize an illness or disease (which essentially constitutes the model of medicine or the approach to medicine) and, accordingly, how to treat illness/disease, reflects the developments in other disciplines, especially social sciences. From this perspective, Engel’s [8] postulate drew on the systems theory and the hierarchical organization of organisms, suggesting that societal and community systems should be simultaneously considered along with more proximal systems, e.g., family, making up the person and the person’s environment. What follows is that human health and illness should be explained in their full contexts, i.e., biologically, psychologically, and socially (hence, the BPS model). Over the years, the model has been both criticized [19] and praised [20], with the most recent trends confirming the validity of the BPS approach [21,22].

Given the advancements made in neurology, genomics, molecular biology, and corresponding debates in social science, it is common today to talk about a more precise ecobiodevelopmental (EBD) framework [23,24,25]. By recognizing neuronal plasticity, the EPD model shows that early life experiences originating in one’s environment, be it stress or any other traumatic event, influence DNA sequencing. This leads to long-lasting effects on the health and well-being of the individual and may be passed on to future generations [24,26]. Accordingly, the EPD model has substantial explanatory power if applied to the study of the implications of early childhood violence exposure and (wartime) trauma [27,28].

Even if the EBD model has substantial explanatory power, given the focus of this paper, it does not lend itself to the purpose of the analysis here. The timeframe that is being applied for the analysis is too short to account for possible alterations in the DNA sequence related to COVID-19-related trauma. Instead, the BPS model will be applied [29,30]. Interestingly, regardless of the growth in the literature either employing the BPS model or addressing the variety of research topics, which the COVID-19 pandemic has prompted, very little attention in the literature has been placed on the biopsychosocial implications of COVID-19. These implications specifically include biological manifestations in the form of respiratory, cardiac, renal, and neurological effects [31]. The social and economic manifestations of COVID-19 were seen in the form of loneliness, social isolation, income, and job insecurities [32]. As for the psychological implications, those who tested positive experienced anxiety, depression, post-traumatic stress disorder (PSD), decreased quality of life, and even death anxiety [33]. There were questions about death anxiety and coping strategies among the survivors. In general, pandemics are known to have psychological and social impacts on people and the biological aspects of their lives [34,35]). Death anxiety was a major psychological issue experienced globally during the COVID-19 pandemic [36].

### 2.2. Anxiety and Coping Strategies in the Context of COVID-19

The literature suggests elevated levels of anxiety concerning COVID-19 and poor mental health associated with the perceived severity of the virus [37]. Fear of death is a central and fundamental experience for humans [38]. Menzies (2012) also suggests that humans have been fighting death anxiety for as long as they have existed. Humans are the only species with the cognitive ability to anticipate death [39]. Indeed, Greenberg’s [40] terror management theory (TMT) is based on Becker’s [38] ideas on death anxiety. The theory ascertains that the fear of death influences the thinking and behavior of people. When death-related thoughts are accessible, people reduce their death anxiety and cope with it by denying their thoughts [41]. Death anxiety is related to coping strategies because of its ambiguous, uncontrollable, and immutable nature [42].

Coping strategies in response to anxiety involve an individual regulating his/her internal and external desires beyond his/her tolerance, through behavioral and cognitive efforts [43]. Two types of coping strategies have been identified [44], i.e., problem-focused and emotion-focused coping. Problem-focused coping involves self- and environment-based direct activities in an attempt to eliminate threatening conditions. Emotion-focused coping involves controlling undesired emotions caused by stressful conditions [44]. When a patient struggles with any illness, they have to deal with its challenges, including biological, physical, emotional, interpersonal, and spiritual. People develop different ways to cope with their fears related to death, which may be adaptive or maladaptive. These coping strategies might include building meaningful relationships and social support. Maladaptive coping strategies in response to a powerful fear of death might include avoidance coping [39].

Current literature suggests that people have adopted different coping strategies to deal with death anxiety during the COVID-19 pandemic. For instance, people suffering from a higher level of death anxiety mostly adopted coping avoidance strategies, including self-distraction, denial, and disengagement [45]. The levels of death anxiety differ across the world and have driven several human behaviors influencing coping mechanisms in daily life [46]. A study was conducted using the BPS model to predict people’s psychological well-being during the pandemic and to investigate what coping strategies they most commonly use. The findings suggest that intentional coping strategies were used by people with high psychological well-being and more passive coping strategies by people with low psychological well-being. The biopsychosocial model suggests that pain, somatic, medical, cognitive, emotional, and behavioral factors should be considered to have a more holistic view of the subjective health phenomenon during the COVID-19 pandemic. Hence, the use of the BPS to study the implications of COVID-19 has been suggested in the literature [47,48].

## 3. Materials and Methods

### 3.1. The Key Concepts, Research Method, and Sampling Strategy

One could argue that ‘COVID-19 survivor’ sounds very dramatic and, hence, a more suitable term should be identified to refer to those individuals who tested positive, were treated at home, suffered from bio-sociopsychological consequences of the virus, and succeeded in coping with the experience. Indeed, in some ways, the word “survivor” is dramatic. In absence of a convincing alternative, this paper upholds the term survivor for two reasons, i.e., (i) there is a tendency in COVID-19 literature to use the word ‘survivor” [49,50]; (ii) in many instances, the circumstances were, indeed, dramatic.

With regard to the research methods—this is a retrospective study involving participants who tested positive for COVID-19 (through rapid tests) and who tested positive for antibodies during the first or second wave of the COVID-19 pandemic. The first wave of the COVID-19 pandemic was between 15 March and 30 June 2020. The second wave of the pandemic was between 1 July and 15 October 2020.

A correlational research design was followed in the present study. A total of 250 participants were approached through two Facebook pages, named “Corona-Survivors in Pakistan” and “Corona Warriors Pakistan”, as well as through WhatsApp and LinkedIn. The response rate was low, as only 100 participants responded and filled out the questionnaires. Accordingly, the sample against which this study was conducted included 100 (53 men and 47 women) COVID-19 survivors aged 20 to 60 (mean age = 30.16, SD =10.27) from the city of Lahore (Pakistan). The non-probability purposive sampling tool place from January to April 2021. The link to the study was circulated through different social media channels, including Facebook, WhatsApp, and LinkedIn. The Google Forms application was used to collect the responses to the survey questions. The survey was conducted in English. Apart from the research-related questions, the survey included the following: the informed consent section, details about the study and its purpose, permission for voluntary participation, a confidentiality–anonymity clause, and a section on demographics. After completing the section on demographic information, participants were directed to the other three sections containing the study measures. The results were collected and calculated through SPSS, version 24.

For the sample recruitment, the following inclusion and exclusion criteria were used to control the confounding variables. Only participants who featured the following characteristics and could, thus, be characterized as survivors were selected to be a part of the study: (a) tested positive for COVID-19 during the first or the second wave of COVID-19, (b) were quarantined after testing positive and received their treatment at home; (c) had a minimum undergraduate education level, which in the context of Pakistan guarantees the ability to comprehend and respond to the survey. In line with the study exclusion criteria, children and adolescents were not included. Similarly, participants diagnosed with mental disorders or physical disabilities have been excluded from the study. The rationale behind these exclusion criteria was to keep the sample homogenous and maintain the controls in the study. Including both adolescents and the adult population in the sample would have been challenging and would likely have presented limitations in the form of the lack of control on the biopsychosocial impacts of COVID-19 on both populations as both have different characteristics. Individuals with either physical disabilities or who were diagnosed with mental illness represent distinct groups of the population, especially regarding the biopsychosocial implications of COVID-19. Lastly, due to the proportionate frequencies of adult populations, it was nearly impossible to seek a comparable number of participants with psychical disabilities and clinical conditions in addition to the general population.

### 3.2. Measures

#### 3.2.1. Demographic Information Sheet

A sheet containing questions related to the personal characteristics of participants was developed, asking about their gender, age, income, educational level, information about the COVID-19 symptoms during the infection period, the treatment and home remedies they took during their illness, details about the period of their COVID-19 diagnoses, and if COVID-19 affected their mental health.

#### 3.2.2. The Biopsychosocial Symptoms Questionnaire

The Biopsychosocial Symptoms Questionnaire assesses COVID-19’s physical, psychological, social, and financial impact on adults. It comprises 21 items answered on a 5-point rating scale ranging from 1 (strongly disagree) to 5 (strongly agree) to determine the impact of COVID-19 across various domains of life. The measure has 4 subscales, namely physical impact (1, 2, 3, 4, 5, 6), psychological impact (7, 8, 9, 10, 11, 12), social impact (13, 14, 15, 16, 17, 18), and financial impact (19, 20, 21). The Biopsychosocial Symptoms Questionnaire has an alpha reliability of 0.89, and its validity score is 0.85. The analysis was conducted through SPSS version 24 [51].

#### 3.2.3. The Death Anxiety Scale

The indigenously developed death anxiety scale measures the behaviors/attitudes of people toward death. This scale is made through a survey and has 23 items with 4 subscales. Acceptance (1, 3, 10, 11, 12, 15, 17, 18, 19, 20), fear (2, 5, 6, 9, 13, 14, 16), neutral acceptance (8, 21, 22, 23), and faith (4, 7) are the subscales of this scale. The participants indicate their agreement with the statement using a 6-point Likert scale including 1 = “Strongly disagree,” 2 = “disagree, “3 = “somehow agree,” 4 = “somehow disagree,” 5 = “agree,” and 6 = “strongly agree”. Its reliability is 0.84, and its validity score is 0.85. A total score can be calculated by adding each item score [52].

#### 3.2.4. The Brief COPE Scale

The measure is a self-reported questionnaire with 28 items that measure how people deal with stressful life events. The questionnaire items include effective and ineffective ways to cope with difficult situations. It has three subscales, namely problem-focused coping (items 2, 7, 10, 12, 14, 17, 23, 25), emotional-focused (items 5, 9, 13, 15, 18, 20, 21, 22, 24, 26, 27, 28), and avoidant-coping (items 1, 3, 4, 6, 8, 11, 16, 19). The statements are answered on a 4-point Likert scale with 1 = I have not been doing this at all, 2 = A little bit, 3 = A medium amount, and 4 = I have been doing this a lot. The following facets of coping are also reported on the scale: self-distraction, denial, substance use, behavioral disengagement, emotional support, venting, humor, acceptance, self-blame, religion, active coping, use of instrumental support, positive reframing, and planning. As Cronbach’s alpha indicates, the scale’s internal consistency is 0.25 to 1.00, and the temporal stability ranges from 0.05 to 1.00 [53].

### 3.3. Statistical Analysis

A descriptive analysis was carried out to estimate the sample’s mean, standard deviation, percentages, and frequencies. For the mediation analysis, inferential statistics were carried out through Pearson correlation coefficients and multiple regression.

## 4. Results

### 4.1. Descriptive Analysis of Study Variables

As shown in Table 1, all participants fall into adulthood and are COVID-19 survivors. Among the 53% of men and 47% of women, according to the results, 50% were unmarried, 48% were married, and 2% were widowed. The participants were asked about the COVID-19 symptoms they suffered when they tested positive, and the reported symptoms were fever, cough, shortness of breath, tiredness, body pain, and sore throat. Only 9% of the people had just one symptom, 8% reported two types of symptoms, 11 % reported three, 25% reported four types, 46% reported five, and only 1 % reported six types of COVID-19 symptoms.

### 4.2. Correlation between Variables

Table 2 shows a significantly strong negative correlation between coping strategies and death anxiety (r = −0.063 *) and between coping strategies and the biopsychosocial symptoms of COVID-19 (r = −0.096). The results also suggest a significant weak positive relationship between death anxiety and biopsychosocial symptomatology of COVID-19 (r = 0.025 **).

Multiple regression on death anxiety is a mediator between biopsychosocial symptoms of COVID-19 and coping strategies among COVID-19 survivors.

**Figure 1:**
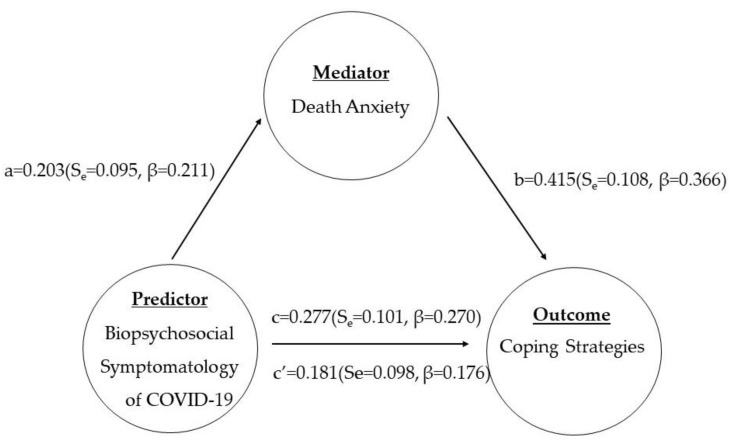
Multiple regression on death anxiety as a mediator between symptomatology and coping strategies among COVID-19 survivors.

In Figure 1 (below), using multiple regression, death anxiety mediates the biopsychosocial symptomatology of COVID-19 and coping strategies among COVID-19 survivors in three steps. In the first step, the ‘coping strategies’ outcome variable was regressed on the predictor biopsychosocial symptomatology to establish an effect to mediate death anxiety (path c). The outcome score was c = 0.277 (Se = 0.101, β = 0.270, *p* = 0.007). In the second step, death anxiety was regressed on the ‘predictor’ biopsychosocial symptomatology variable to establish (path a) a = 0.203 (Se = 0.095, β = 0.21, *p* = 0.035). In the last step, the coping strategies score was regressed on both the predictor biopsychosocial symptomatology and the death anxiety mediator (path b and c’) b = 0.415(Se = 0.108, β = 0.366, *p* = 0.00) and c’ = 0.181(Se = 0.098, β = 0.176, *p* = 0.04). The results indicate that death anxiety partially mediates the relationship between biopsychosocial symptomatology and coping strategies as c’ < c and c’ are not equal to 0.

## 5. Discussion

This study explained the relationship between the biopsychosocial symptomatology of COVID-19, death anxiety, and coping strategies among COVID-19 survivors. It was indicated in a systematic review that medical history and social and psychological factors were associated with mental illnesses (such as death anxiety) during the COVID-19 pandemic [54]. Increased prevalence of death anxiety in public during the pandemic could be attributed to the biopsychosocial factors related to COVID-19 [55]. It was also suggested that during the pandemic, death anxiety was highly associated with coping strategies [56].

The current study’s finding suggests a significantly strong negative relationship between coping strategies and biopsychosocial symptomatology of COVID-19 and death anxiety among COVID-19 survivors. In line with this, previous literature studies from other parts have confirmed these results. Nia et al. [57] found a negative relationship between coping strategies and death anxiety. Research suggests a negative correlation between death anxiety and coping strategies, and people with more religious coping strategies have lower death anxiety [58]. Moreover, a negative relationship between coping strategies and biopsychosocial symptomatology of COVID-19 has been established [59].

Furthermore, the present study’s findings suggest a weak positive association between the biopsychosocial symptomatology of COVID-19 and death anxiety. In line with this, a positive relationship between biopsychosocial symptomatology and death anxiety among cancer patients has been confirmed [60]. This shows that people facing advanced cancer symptoms had higher death anxiety, establishing a connection between death anxiety and severe health conditions.

The findings of this study also suggest that death anxiety is a mediator in the relationship between the biopsychosocial impacts of COVID-19 and coping strategies among COVID-19 survivors. When people witnessed their relatives becoming infected by the virus, their death-related fears increased [61]. They also feared the social outcome of becoming infected by the virus, as the pandemic has affected society in the form of unemployment and economic inflation. COVID-19-infected individuals were also more worried about the treatment expenses they had to bear for their recovery, along with death anxiety [62]. There is a connection between death anxiety and many psychological disorders. Due to this interplay, it is known that death anxiety is related to coping strategies due to its ambiguous, uncontrollable, and immutable nature [42]. Therefore, it can be concluded that death anxiety is a mediator in the relationship between the biopsychosocial symptoms of COVID-19 and coping skills. In line with these findings is the research by [63] on breast cancer in women; they investigated death anxiety as a mediator in the relationship between self-compassion and depression. From the study findings, it was concluded that death anxiety is a mediator between illness symptoms and coping strategies in people suffering from medical illnesses.

The literature suggests that people have used different coping strategies during pandemics to deal with their challenges [64]. People who have successfully dealt with their challenges used adaptive coping strategies. Regarding coping strategies used during the COVID-19 pandemic, problem-focused coping, social support seeking, avoidance coping, and positive appraisal of the situation have been used to cope with the fear of death, anxiety, and depression [64]. Moreover, it was suggested that people use online psychotherapeutic approaches to address biopsychosocial issues during the pandemic [65,66].

As a correlational study design was used in this study, it limited the conclusion to be drawn on a cause-and-effect basis. One other shortcoming of the present study is that the participants were only adults, thus limiting the generalizability of findings to children and adolescents. Another limitation of the study is that the researchers had a limited time to complete the work, limiting the sample size due to time constraints. Furthermore, due to strict lockdown measures implemented in Pakistan during the COVID-19 pandemic, data collection by physically approaching the participants was not possible. This study has the limitation of a small sample size as participants were approached online. Even if the group of potential respondents was sizeable at first, many of the individuals who were initially inclined to join the study ultimately did not respond. This resulted in a low response rate, limiting the sample size. Moreover, it took time to receive consent from COVID-19 survivors to participate in the study, and many refused to participate in the study precisely on this account. As part of future endeavors, studies should include different demographic variables that can impact the associations between these study variables. Studies should be conducted with other populations and from other regions of Pakistan with increased sample sizes to improve the generalizability of the results.

## 6. Conclusions

This paper investigated the coping strategies of COVID-19 survivors in response to the biopsychosocial implications of COVID-19. The BPS model was applied. Should the necessary prerequisites be met, it would be interesting to conduct an EBD-informed analysis of the longitudinal implications of COVID-19 on selected groups of the population, e.g., first-grade pupils. It would be equally interesting to use the research method employed in this study to examine populations in different countries and, provided all requirements are met, compare the results. Similarly, it would be very useful to examine the populations excluded from the scope of this study, including individuals with disabilities, adolescents, and children. Insight into the experiences of the groups and ways of dealing with the COVID-19 pandemic would be invaluable in terms of providing better healthcare and therapy to respective individuals.

## Figures and Tables

**Table 1:** Demographic characteristics of the participants (N = 100).

Variables	F	%
Gender		
Male	53	52.5
Female	47	46.5
Marital Status		
Married	48	47.5
Unmarried	50	49.5
Widow	2	2.0
Number of symptoms experienced by COVID-19 survivors		
One symptom	9	8.9
Two symptoms	8	7.9
Three symptoms	11	10.9
Four symptoms	25	24.8
Five symptoms	46	45.5
Six symptoms	1	1.0

**Table 2:** Correlation among study variables (N = 100).

Variables	Coping Strategies	Death Anxiety	Biopsychosocial Symptoms of COVID-19
Coping strategies	1	−0.063 *	−0.096
Death anxiety		1	0.025 **
Biopsychosocial symptoms of COVID-19			1

## Data Availability

Should you wish to obtain all data pertaining to this research, please contact the corresponding author.

## References

[1] Anderson M., Mckee M., Mossialos E. (2020). Developing a sustainable exit strategy for COVID-19: Health, economic and public policy implications. J. R. Soc. Med..

[2] Parry L.J., Asenbaum H., Ercan S.A. (2021). Democracy in flux: A systemic view on the impact of COVID-19. Transform. Gov. People Process Policy.

[3] Renda A. (2022). COVID-19 and privacy: A European dilemma. Digit. Policy Regul. Gov..

[4] Kundu S., Latif M., Hořejší P., Visvizi A., Troisi O., Grimaldi M. (2023). Using DES to Improve the Efficiency of a COVID-19 Vaccination Centre. RIIFORUM 2022: Research and Innovation Forum 2022.

[5] Wang J., Peng Y., Xu H., Cui Z., Williams R.O. (2020). The COVID-19 Vaccine Race: Challenges and Opportunities in Vaccine Formulation. AAPS Pharmscitech.

[6] Ferreras-Garcia R., Sales-Zaguirre J., Serradell-López E., Visvizi A., Troisi O., Grimaldi M. (2023). Generic Competences in Higher Education after COVID-19 Pandemic. RIIFORUM 2022: Research and Innovation Forum 2022.

[7] Daniela L., Visvizi A. (2021). Remote Learning in Times of Pandemic: Issues, Implications and Best Practice.

[8] Engel G.L. (1977). The need for a new medical model: A challenge for biomedicine. Science.

[9] Wainwright T.W., Low M. (2020). Why the biopsychosocial model needs to be the underpinning philosophy in rehabilitation pathways for patients recovering from COVID-19. Integr. Healthc. J..

[10] Galbadage T., Peterson B.M., Wang D.C., Wang J.S., Gunasekera R.S., Richard S. (2020). Biopsychosocial and Spiritual Implications of Patients With COVID-19 Dying in Isolation. Front. Psychol..

[11] Kop W.J. (2021). Biopsychosocial Processes of Health and Disease During the COVID-19 Pandemic. Psychosom. Med..

[12] Guaracha-Basáñez G.A., Contreras-Yáñez I., Hernández-Molina G., Estrada-González V.A., Pacheco-Santiago L.D., Valverde-Hernández S.S., Galindo-Donaire J.R., Peláez-Ballestas I., Pascual-Ramos V. (2022). Quality of life of patients with rheumatic diseases during the COVID-19 pandemic: The biopsychosocial path. PLoS ONE.

[13] Brailovskaia J., Margraf J. (2021). The relationship between burden caused by coronavirus (COVID-19), addictive social media use, sense of control and anxiety. Comput. Hum. Behav..

[14] Brailovskaia J., Truskauskaite-Kuneviciene J., Margraf J., Kazlauskas E. (2021). Coronavirus (COVID-19) outbreak: Addictive social media use, depression, anxiety and stress in quarantine—An exploratory study in Germany and Lithuania. J. Affect. Disord. Rep..

[15] Güldal Ş., Kılıçoğlu N., Kasapoğlu F. (2022). Psychological Flexibility, Coronavirus Anxiety, Humor and Social Media Addiction During COVID-19 Pandemic in Turkey. Int. J. Adv. Couns..

[16] Qi R., Chen W., Liu S., Thompson P.M., Zhang L.J., Xia F., Cheng F., Hong A., Surento W., Luo S. (2020). Psychological morbidities and fatigue in patients with confirmed COVID-19 during disease outbreak: Prevalence and associated biopsychosocial risk factors. medRxiv.

[17] Lahav Y. (2020). Psychological distress related to COVID-19—The contribution of continuous traumatic stress. J. Affect. Disord..

[18] Tuason M.T., Güss C.D., Boyd L. (2021). Thriving during COVID-19: Predictors of psychological well-being and ways of coping. PLoS ONE.

[19] McLaren N. (1998). A Critical Review of the Biopsychosocial Model. Aust. N. Z. J. Psychiatry.

[20] Suls J., Rothman A. (2004). Evolution of the Biopsychosocial Model: Prospects and Challenges for Health Psychology. Health Psychol..

[21] Ghaemi S. (2009). The rise and fall of the biopsychosocial model. Br. J. Psychiatry.

[22] Adler R.H. (2009). Engel’s biopsychosocial model is still relevant today. J. Psychosom. Res..

[23] Kelly M.M., Li K. (2019). Poverty, Toxic Stress, and Education in Children Born Preterm. Nurs. Res..

[24] Kliegman R.M., Kliegman R.M., Geme J. (2019). Developmental and Behavioral Theories. Nelson Textbook of Pediatrics.

[25] Huang C.-C., Chen Y., Cheung S. (2021). Early childhood exposure to intimate partner violence and teen depression symptoms in the U.S. Health Soc. Care Community.

[26] Garner A.S. (2016). Thinking Developmentally: The Next Evolution in Models of Health. J. Dev. Behav. Pediatr..

[27] Herman-Smith R. (2013). Intimate Partner Violence Exposure in Early Childhood: An Ecobiodevelopmental Perspect. Health Soc. Work..

[28] Voith L.A., Hamler T., Francis M.W., Lee H., Korsch-Williams A. (2020). Using a Trauma-Informed, Socially Just Research Framework with Marginalized Populations: Practices and Barriers to Implementation. Soc. Work. Res..

[29] Banerjee D., Kosagisharaf J.R., Sathyanarayana Rao T.S. (2021). ‘The dual pandemic’ of suicide and COVID-19: A biopsychosocial narrative of risks and prevention. Psychiatry Res..

[30] Ali J.S. (2020). COVID-19 pandemic is a worldwide typical Biopsychosocial crisis. J. Ideas Health.

[31] Bourgonje A.R., Abdulle A.E., Timens W., Hillebrands J.L., Navis G.J., Gordijn S.J., Bolling M.C., Dijkstra G., Voors A.A., Osterhaus A.D. (2020). Angiotensin-converting enzyme 2 (ACE2), SARS-CoV-2 and the pathophysiology of coronavirus disease 2019 (COVID-19). J. Pathol..

[32] Wong S.Y.S., Zhang D., Sit R.W.S., Yip B.H.K., Chung R.Y., Wong C.K.M., Chan D.C.C., Sun W., Kwok K.O., Mercer S.W. (2020). Impact of COVID-19 on loneliness, mental health, and health service utilization: A prospective cohort study of older adults with multimorbidity in primary care. Br. J. Gen. Pract..

[33] Méndez R., Balanzá-Martínez V., Luperdi S.C., Estrada I., Latorre A., González-Jiménez P., Feced L., Bouzas L., Yépez K., Ferrando A. (2021). Short-term neuropsychiatric outcomes and quality of life in COVID-19 survivors. J. Int. Med..

[34] Monaghan L.F. (2020). Coronavirus (COVID-19), pandemic psychology and the fractured society: A sociological case for critique, foresight and action. Sociol. Health Illn..

[35] Waters L., Algoe S.B., Dutton J., Emmons R., Fredrickson B.L., Heaphy E., Moskowitz J.T., Neff Niemiec K.R., Pury C., Steger M. (2022). Positive psychology in a pandemic: Buffering, bolstering, and building mental health. J. Posit. Psychol..

[36] Spoorthy M.S., Pratapa S.K., Mahant S. (2020). Mental health problems faced by healthcare workers due to the COVID-19 pandemic—A review. Asian J. Psychiatry.

[37] Li J.B., Yang A., Dou K., Cheung R.Y.M. (2020). Self-control moderates the association between the perceived severity of the coronavirus disease 2019 (COVID-19) and mental health problems among the Chinese public. Int. J. Environ. Res. Public Health.

[38] Becker E. (1973). The Denial of Death.

[39] Menzies R.E., Menzies R.G., Iverach L. (2018). Curing the Dread of Death: Theory, Research and Practice.

[40] Greenberg J., Solomon S., Pyszczynski T. (1997). Terror Management Theory of Self-Esteem and Cultural Worldviews: Empirical Assessments and Conceptual Refinements. Adv. Exp. Soc. Psychol..

[41] Shakil M., Ashraf F., Muazzam A., Amjad M., Javed S. (2021). Work status, death anxiety, and psychological distress during COVID-19 pandemic: Implications of the terror management theory. Death Stud..

[42] Witkowski J. (2016). Coping and attitudes toward dying and death in German adults. OMEGA J. Death Dying.

[43] Abedini S.M., Montazeri S., Khalatbari J. (2012). Comparison between Styles of Coping with Stress in Patients with Multiple Sclerosis and Healthy people in the East of Mazandaran. J. Maz. Univ. Med. Sci..

[44] Lazarus R., Folkman S. (1985). Coping and Adaptation.

[45] Partouche-Sebban J., Vessal S.R., Sorio R., Castellano S., Khelladi I., Orhan M.A. (2021). How death anxiety influences coping strategies during the COVID-19 pandemic: Investigating the role of spirituality, national identity, lockdown, and trust. J. Mark. Manag..

[46] Menzies R.E., Menzies R.G. (2020). Death anxiety in the time of COVID-19: Theoretical explanations and clinical implications. Cogn. Behav. Ther..

[47] Griffith D.M., Sharma G., Holliday C.S., Enyia O.K., Valliere M., Semlow A.R., Stewart E., Blumenthal R.S. (2020). Men and COVID-19: A biopsychosocial approach to understanding sex differences in mortality and recommendations for practice and policy interventions. Prev. Chronic Dis..

[48] Leonardi M., Lee H., Van der V.S., Maribo T., Cuenot M., Simon L., Paltamaa J., Maart S., Tucker C., Besstrashnova Y. (2020). Avoiding the banality of evil in times of COVID-19: Thinking differently with a biopsychosocial perspective for future health and social policies development. SN Compr. Med..

[49] Fernández-de-las-Peñas C., Palacios-Ceña D., Gómez-Mayordomo V., Florencio L.L., Cuadrado M.L., Plaza-Manzano G., Navarro-Santana M. (2021). Prevalence of post-COVID-19 symptoms in hospitalized and non-hospitalized COVID-19 survivors: A systematic review and meta-analysis. Eur. J. Intern. Med..

[50] Mazza M.G., De Lorenzo R., Conte C., Poletti S., Vai B., Bollettini I., Melloni E.M.T., Furlan R., Ciceri F., Rovere-Querini P. (2020). Anxiety and depression in COVID-19 survivors: Role of inflammatory and clinical predictors. Brain Behav. Immun..

[51] Naseem F. (2020). Biopsychosocial Symptoms Questionnaire. Unpublished. Master’s Thesis.

[52] Liaqat A. (2019). Development of Death Anxiety Scale.

[53] Carver C.S. (1997). You want to measure coping, but your protocol’s too long: Consider the brief COPE. Int. J. Behav. Med..

[54] Vindegaard N., Benros M.E. (2020). COVID-19 pandemic and mental health consequences: A systematic review of the current evidence. Brain Behav. Immun..

[55] Zhang Q., Zheng R., Fu Y., Mu Q., Li J. (2021). Mental health consequences during alerting situations and recovering to a new normal of coronavirus epidemic in 2019: A cross-sectional study based on the affected population. BMC Public Health.

[56] Özgüç S., Kaplan S.E., Tanriverdi D. (2021). Death anxiety associated with coronavirus (COVID-19) disease: A systematic review and meta- analysis. Omega.

[57] Nia H.S., Soleimani M.A., Ebadi A., Taghipour B., Zera’Tgar L., Shahidifar S. (2017). The relationship between spiritual intelligence, spiritual well-being, and death anxiety among Iranian veterans. J. Mil. Med..

[58] Freh F.M., Chung M.C. (2021). Post-traumatic stress disorder and death anxiety among Iraqi civilians exposed to a suicide car bombing: The role of religious coping and attachment. J. Ment. Health.

[59] Ghanem I., Castelo B., Jimenez-Fonseca P., Carmona-Bayonas A., Higuera O., Beato C., García T., Hernández R., Calderon C. (2019). Coping strategies and depressive symptoms in cancer patients. Clin. Transl. Oncol..

[60] Loughan A.R., Husain M., Ravyts S.G., Willis K.D., Braun S.E., Brechbiel J.K., Aslanzadeh F.J., Rodin G., Svikis D.S., Thacker L. (2021). Death anxiety in patients with primary brain tumor: Measurement, prevalence, and determinants. Palliat. Support. Care.

[61] Joaquim R.M., Pinto AL C.B., Guatimosim R.F., de Paula J.J., Costa D.S., Diaz A.P., da Silva A.G., Pinheiro M.I., Serpa A.L., Miranda D.M. (2021). Bereavement and psychological distress during COVID-19 pandemics: The impact of death experience on mental health. Curr. Res. Behav. Sci..

[62] Fereidooni R., Mootz J., Sabaei R., Khoshnood K., Heydari S.T., Moradian M.J., Taherifard E., Nasirian M., Vardanjani H.M. (2021). The COVID-19 pandemic, socioeconomic effects, and intimate partner violence against women: A population-based cohort study in Iran. SSRN Electron. J..

[63] Sheida S.S., Ahmad A., Alireza A., Reza M.M., Bagher G.B. (2019). Relationship of Attachment Styles to God and Depression with Death Anxiety as a Mediator among Women with Breast Cancer. Relig. Health.

[64] Salopek-Žiha D., Hlavati M., Gvozdanović Z., Gašić M., Placento H., Jakić H., Klapan D., Šimić H. (2020). Differences in distress and coping with the COVID-19 stressor in nurses and physicians. Psychiatr. Danub..

[65] Tanhan A., Yavuz K.F., Young J.S., Nalbant A., Arslan G., Yıldırım M., Çiçek İ. (2020). A Proposed Framework Based on Literature Review of Online Contextual Mental Health Services to Enhance Wellbeing and Address Psychopathology During COVID-19. Electron. J. Gen. Med..

[66] Mukaetova-Ladinska E.B., Kronenberg G. (2021). Psychological and neuropsychiatric implications of COVID-19. Eur. Arch. Psychiatry Clin. Neurosci..

